# Effects of arthroscopic-assisted surgery on irreducible developmental dislocation of hip by mid-term follow-up: An observational study

**DOI:** 10.1097/MD.0000000000004601

**Published:** 2016-08-19

**Authors:** Hui-fa Xu, Ya-bo Yan, Chao Xu, Tian-qing Li, Tian-feng Zhao, Ning Liu, Lu-yu Huang, Chun-li Zhang, Wei Lei

**Affiliations:** Department of Orthopeadics, Xijing Hospital, Fourth Military Medical University, Xi’an, People's Republic of China..

**Keywords:** acetabuloplasty, arthroscope, developmental dislocation of the hip, irreducible, mid-term clinical effect

## Abstract

The purpose of this study was to investigate the indications, surgical technique, and the clinical effects of arthroscopic-assisted treatment of irreducible developmental dislocation of the hip by mid-term follow-up. Arthroscopic-assisted surgeries were performed on 40 children (52 hips) between January 2005 and December 2009. Anterior and antero-superior greater trochanter portals were used in these treatments. Spica cast and abduction splint were applied for 3 months postoperatively. The follow-up was conducted on every 3 months postoperatively. During 12-month follow-up, a secondary treatment such as acetabuloplasty and/or femoral osteotomy (shortening, varus, and derotation) was applied if the acetabular angle was greater than 25°. The pelvic acetabular angle, Mckay and Severin score were evaluated every 6 months in all children. With 36 to 96 months (average 71 months) follow-up, 35 children (44 hips) were successfully followed up with complete case data while 5 children unsuccessfully. According to Tönnis classification, there were 5 grade 1 hips, 14 grade 2 hips, 14 grade 3 hips, 11 grade 4 hips, in which 3 children (4 hips) were failed in arthroscopic reduction and femoral head avascular necrosis occurred in 2 children (4 hips). According to Mckay standard, the good rate is 100%. According to Severin standard, the good rate is 84.1%. Arthroscopic assisted treatment is an effective way of reduction of the irreducible hip. Compared with the open reduction, arthroscopic treatment combined with acetabuloplasty and/or femoral osteotomy has advantages of less trauma and better function preservation.

## 1 Introduction

Developmental dislocation of the hip (DDH), with a reported incidence of 0.9‰ to 35‰, is a very common disease in pediatric orthopedics.^[1–4]^ Children over 18 months or who failed in close reduction are treated through open reduction, such as pulvinar resection, acetabuloplasty, and femoral osteotomy. Nevertheless, the most vital risk of open reduction is femoral head avascular necrosis caused by several factors, of which the main one is the damage of artery ring by the increasing pressure on femoral head and the open reduction.^[5]^ It is reported that the incidence rate of femoral head avascular necrosis of open reduction on DDH postoperatively is 0% to 69%.^[6–8]^ In order to treat the irreducible DDH earlier and reduce the complications of open reduction, the arthroscopic treatment becomes an alternative way in recent years.^[9–12]^ However, the report on this method is of few cases and fewer follow-ups. The purpose of this study was to investigate the indications, surgical technique, and the clinical effects of arthroscopic-assisted treatment of irreducible developmental dislocation of the hip by mid-term follow-up.

## 2 Materials and methods

### 2.1 Patients

From January 2005 to December 2009, arthroscopic reductions with pulvinar resection, ligamentum teres excision, transverse acetabular ligament release, and labrum fixation under arthroscopes were performed on 40 children (52 hips). Thirty-five children (44 hips, 20 on right and 24 on left, and 26 single and 9 bilateral) of 9 boys and 26 girls, aged from 4 to 40 months (17.7 months on average), were successfully followed up for 36 to 96 months (average 71 months). According to Tönnis classification, there were 5 grade 1, 14 grade 2, 14 grade 3, and 11 grade 4 hips. All patients, who were of first-time consultancy, were failed in the close reduction. Children lied on supine position and hip and knees were bent to 90°. Thumbs were placed at inner thigh, index finger and middle finger were placed at great trochanter, and the abduction and extorsion were conducted on bilateral thighs. When the hip abduction angle was less than or equal to 70°, the abduction was limited and the adductor was of an arch, and then adductor tendon was cut off under general anesthesia. Close reduction was difficult to achieve in some patients, whose Ortolani tests were negative, and some close reduction was successful but the reduction could not be sustained by spica cast.

## 3 Methods

### 3.1 Portals

**Figure 1:**
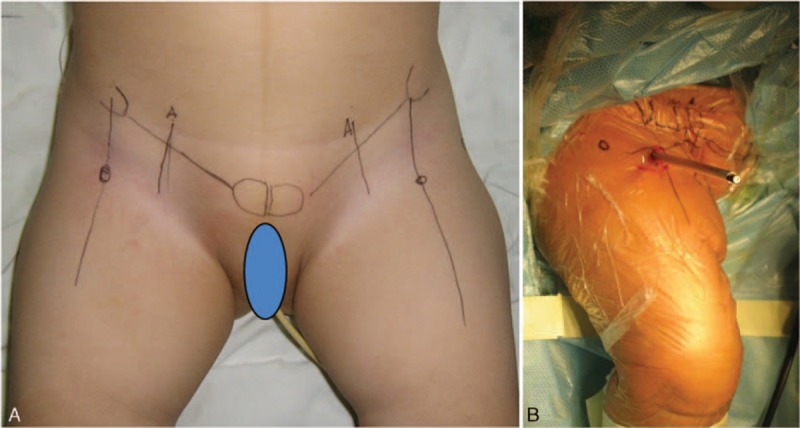
A, The puncture point of anterior portal was at the intersection of the anterior superior iliac spine perpendicular and the pubic symphysis horizontal line. B, A cannula was inserted with the sagittal plane of 45° upward and coronal plane of 45° inward.

Anterior and antero-superior great trochanter portals were applied in these treatments. The puncture point of anterior portal was at the intersection of the anterior superior iliac spine perpendicular and the pubic symphysis horizontal line with the sagittal plane of 45° upward and coronal plane of 45° inward. The antero-superior great trochanter portal was constructed under the arthroscope (Fig. 1).

### 3.2 Surgical technique

**Figure 2:**
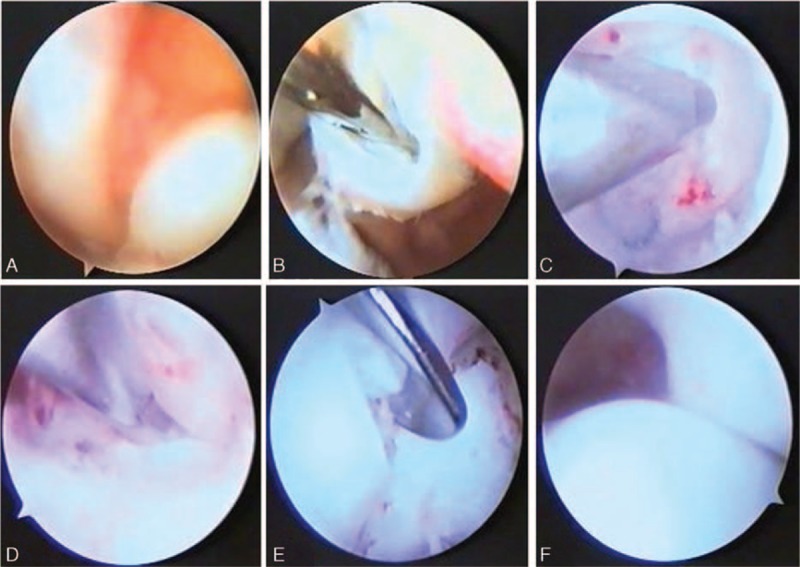
A, Ligamentum teres connected with femoral head were confirmed. B, Ligamentum teres were excised by arthroscopy planning tool. C, All pulvinars in acetabulum were removed. D, The transverse acetabular ligament was cut off. E, The labrum was incised by hooked blade radially. F, Reduction of the femoral head.

All the surgical treatments were under general anesthesia with close reduction first and arthroscopic treatments were performed after the failure of the close reduction. The surgery was performed in supine position with the affected hip elevated and the adductor tendons of 35 children (44 hips) were cut off. The arthroscopy was performed using a 4 mm cannulated arthroscopic instrument with a 30° scope. According to the preoperation position mark, size-18 needle was inserted into the puncture point and normal saline was injected to extend the joint. With a 1 cm incision, blunt dissection was used to puncture into the hip joint for examination. The antero-superior great trochanter portal was set with the assistant of the arthroscope. The scope was turned and rotated sequentially to conduct full examination and then femoral head dislocation, hypertrophic ligamentum teres, acetabulum filled with pulvina, posterior labrum vulgus, slight anterior labrum varus and regular circle shape of femoral head were visible. Femoral head ligamentum teres and pulvinar were excised and posterior labrum was vaporized and fixed to relieve the obstacles for reduction. Transverse acetabular ligament was cut off and reduction was applied after hemostasis. The thing to note was that posterior two-third of outer-rim incision of the labrum was to keep the integrity of the inner-rim. Joint was washed, reduction was applied on femoral head, and then the arthroscopic treatment was finished. Two arthroscopic incisions were stitched, adductor muscle tenotomy was carried out, and then spica cast was used (Fig. 2, surgical technique under arthroscope).

### 3.3 Postoperative treatment

The improved hip frog cast was applied for 3 months. Afterward, the spica cast was performed for another 3 months and then abduction brace was used for 3 to 6 months. The study was approved by the Ethical Committee of our university. Parents or guardians signed written informed consent for the patients to participate in the study.

### 3.4 Evaluations

The clinical and imaging data were collected and analyzed. Dislocation level was evaluated by Tönnis classification.^[13]^ The results were evaluated by Mckay^[14]^ and Severin standard^[15]^ for the clinical function.

## 4 Results

All the children were followed up for 36 to 96 months (average 71 months), in which 35 children (44 hips) were successfully followed up with complete case data and 5 children unsuccessfully.

The average operation time was 28 minutes (22–36 minutes). Till the end of the follow-up, according to Mckay standard, 35, 9, 0, and 0 hips were rated excellent, good, fine, and poor respectively with 100% good rate. According to Severin standard, 27, 10, 4, 3, 0, and 0 hips were rated I, II, III, IV, V, and VI, respectively, with 84.1% good rate.

**Figure 3:**
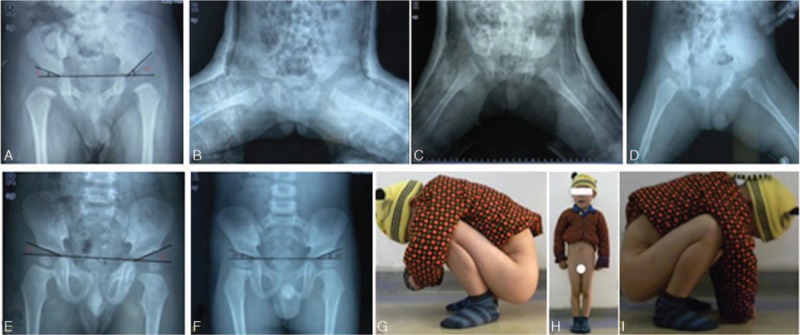
A, 11 months, L: Tönnis II, AC angle: 42°/25°. B, Plaster cast after excision and reduction. C, Plaster cast: 30° in abduction position. D, Abduction brace afterwards. E, Continuous Shenton line with AC angle: 25°/20° after 1 year. F, AC angle: 20°/18° in 42 months. G–I, Excellent (Mckay standard).

**Figure 4:**
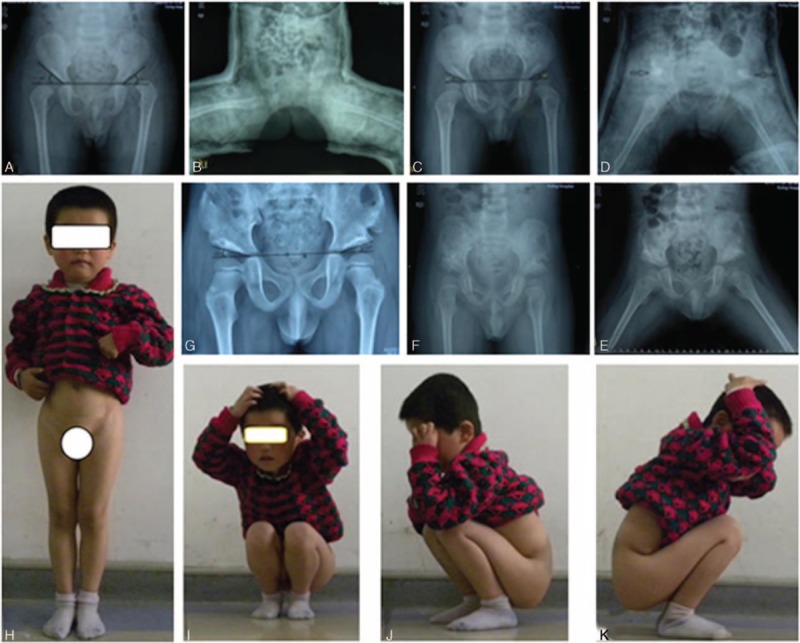
A, 22 months; Tönnis III bilaterally; AC angle: 45°/40°. B, Plaster cast after reduction. C, Good reduction (1 year); AC angle: 28°/27°. D, Pemberton acetabuloplasty. E, Abduction brace (the 6th week). F, Good relationship of femoral head, and acetabulum. G, AC angle: 18°/15° (the 42th month). H–K, Excellent (Mckay standard).

The arthrosopic treatment was performed successfully on 32 cases (40 hips) with follow-up without any redislocation. One case shown in Fig. 3 was symbolic for 13 cases (cases 2, 5, 6, 8, 10, 15, 17, 21, 23, 25, 28, 31, and 34 in Table 1), in which the reduction was successful and the acetabulum was well developed after arthroscopic surgery with the AC angle less than 25° (Fig. 3). The preoperative acetabular angle was 31° to 55° (43.8° on average) and the rectified acetabular angle was 22° to 41° (29.5° on average) in 1-year follow-up. The Pemberton acetabuloplasty^[16]^ was applied on 26 hips of 19 cases (cases 3, 4, 7, 9, 11, 12, 13, 14, 16, 18, 20, 22, 24, 26, 27, 29, 32, 33, and 35 in Table 1) after 13.2 (11–16.5) months of the surgery (Fig. 4) whose acetabulor angle was greater than 25° and the acetabular angle was rectified to 15° to 25° (20.3° on average) till the last follow-up. An additional varus osteotomy was carried out when the Pemberton acetabuloplasty was applied and the neck shaft angle was rectified to 135° with good femoral head containment in 1 case with the neck shaft angle over 160° in poor femoral head containment. Another child (1 hip) was discovered with poor femoral head containment, discontinous Shenton line, and good femoral head containment in internal rotational condition in 1-year follow-up.

**Table 1:**
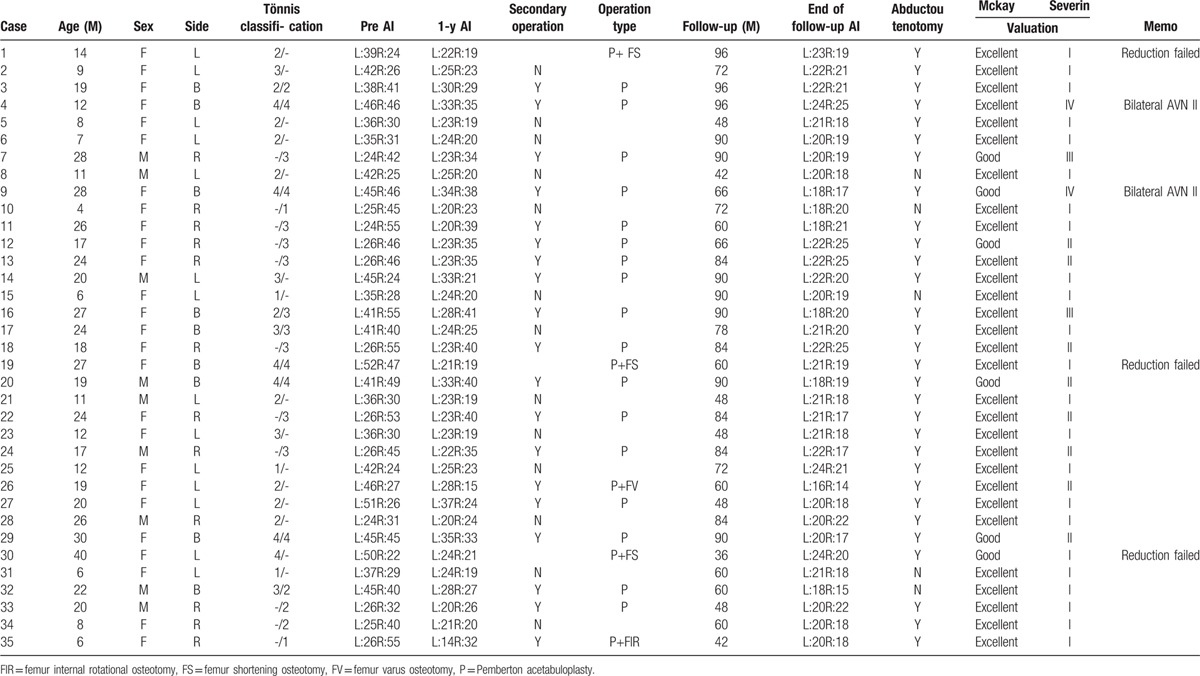
Detailed data of 35 children (44 hips).

Three cases (4 hips) were failed in reduction under arthroscope, of which the labrum of the first case (1 hip) was fully incised radially, so that the hip joint was not stable and the plaster cast could not help to keep stable. Then the secondary open reduction was applied 1 month later. In other 2 cases (3 hips) of grade 4 Tönnis with severe hip dislocation, even after all the treatments under arthroscope, the reduction still could not be achieved. Then the secondary open reduction was applied.

Femoral head avascular necrosis of grade II was found in 2 cases (4 hips) according to MacEwen classification.^[17]^ Among these 2 cases (4 hips), 1 case (2 hips) was diagnosed AVN 6 months after the surgery, and the other case (2 hips) was diagnosed AVN 14 months after the surgery. Both were discovered with slightly limited squat, a bit claudicant, negative Trendelenburg, slight hip abduction, and external rotation, as well as no hip pain while walking and no lower limbs discrepancy.

## 5 Discussions

Developmental dislocation of the hip is one of the common deformities in children, to which the therapy strategies are mainly based on the age of the patient. Most researchers suggest wearing Pavlik for children less than 6 months, manipulative reduction and plaster cast for children aged between 6 months and 18 months,^[18,19]^ and open reduction and acetabuloplasty for children older than 18 months of walking age. However, for all the therapy strategies listed above, it is critical that femoral head needs to be set inside acetabular fossa by reduction. It is reported that the success rate of wearing Pavlik to cure DDH is 74% to 96%.^[20–24]^ To children older than 6 months, manipulative reduction and spica cast are used generally. According to the report by Druschel et al,^[25]^ magnetic resonance imaging scanning used to examine the femoral head containment after the close reduction showed that 22 hips were stable and 27 hips were not. For irreducible children who failed in traditional therapy and with poor containment, open reduction is the only way; whereas open reduction has effects of operation trauma and complications, especially femoral avascular necrosis and limited joint mobility. The main purpose of open reduction method for the irreducible DDH is to remove all the obstacles preventing the reduction. With the development of the arthroscope equipment and technique, the use of hip arthroscopy is developed as well.^[26–30]^ Arthroscopy is used to explore, confirm, and remove all the obstacles. The study on the arthroscopic treatment for irreducible DDH is to verify the followings:

1. whether all the obstacles preventing reduction can be fully eliminated to achieve reduction by arthroscopy
2. whether the acetabular angle can be stimulated to develop normally after arthroscopic reduction
3. the causes for femoral avascular necrosis through the arthroscopic reduction and acetabuloplasty on different therapy stages
4. whether arthroscopic reduction can help to cut down the destruction of femoral head blood supply and reduce the acetabular avascular necrosis

A few successful arthroscopic reductions for DDH have been reported. McCarthy and MacEwen^[31]^ have reported 3 cases with the average age of 14 months, of which 1 case needed a secondary operation because of the permanent acetabular dysplasia. Öztürk et al^[12]^ have reported 9 cases younger than 18 months with arthroscopic treatment and 1 child of 16 months is discovered acetabular dysplasia and Salter innominate osteotomy is required. Eberhardt et al^[11]^ have reported 9 cases of arthroscopic reduction and acetabuloplasty for the treatment of DDH of walking age younger than 18 months, and considered this technique is a new method for DDH. Therefore, it could be an alternative to open reduction of DDH that is classified as grade 2 and 3 dislocations according to Tönnis.

Anterior and antero-superior great trochanter portals were used in these treatments. The medial portal is considered the most prevalent one in DDH treatment by Ludloff et al. The psoas tendon is easily internal rotational proximal femur osteotomytenotomied by this portal, while the acetabular roof cartilage, ligamentum teres, and pulvinar are less visible. Most importantly, medial femoral circumflex artery ring can be hurt easily through medial portal, which can cause high acetabular avascular necrosis.

One-year follow-up showed that the preoperative acetabular angle was 43.8° (31°–55°) and the rectified acetabular angle was 29.5° (22°–41°) in the cases, aged from 4 months to 40 months. The Pemberton acetabuloplasty was applied on 19 cases whose acetabulor angle was greater than 25° and the acetabular angle was rectified to 20.3° (15°–25°) till the last follow-up.

It is known that with the increase of the age, the developmental potential of acetabular cartilage will reduce gradually. Reduction under arthroscopy was successfully performed on 32 children (40 hips). During the 1-year follow-up, 4 children (5 hips) of 15 children (16 hips) under 18 months, and 15 children (21 hips) of 17 children (24 hips) older than 18 months showed acetabular dysplasia. It can be concluded that: Arthroscopic reduction can help to stimulate the development of acetabular roof cartilage for 3 reasons. First, the normal position of femoral head and acetabulum can help to stimulate acetabular cartilage effectively. Second, it can help to increase the blood supply of the hip joint. Schoenecker et al^[32]^ discovered that the blood flow of dysplastic hip is 40% less than the normal hip. Third, the removal of the obstacles in acetabulum can help to expand the capsule space and reduce the articular cavity pressure. Besides, whether acetabuloplasty is performed to rectify the acetabular dysplasia or not should depend on the age and severity of preoperative acetabular dysplasia. Age is not the only factor to consider and not all the children need the treatment of acetabuloplasty. The acetabulum form in femoral head reset to stimulate the acetabulum was more suitable to femoral head than the femur osteotomy, and OA may be delayed by this as well, which of course needs the longer follow-ups. It is believed that arthroscopic reduction and acetabuloplasty should be performed in different periods, which is not consistent with Eberhardt's^[11]^ opinion.

In the series, 2 cases (4 hips) were discovered femoral avascular necrosis of grade II according to MacEwen classification^[17]^ during the follow-up. The avascular necrosis rate of Cashman et al^[33]^ who used Pavlik for the treatment of DDH is 1%. Close reduction is performed on 28 hips aged from 1 to 11 months by Tiderius et al^[34]^ and the avascular necrosis rate is 50% by magnetic resonance imaging examination. Bian and Guo^[18]^ have reported that 39 in 106 hips treated with close reduction and plaster cast for DDH are discovered avascular necrosis with the rate of 36.8%. The report about the avascular necrosis rate is different in open reduction for DDH. Pospischill et al^[6]^ have reported that 64 cases (78 hips) are treated from 1998 to 2007 with a follow-up of 6.8 years and the avascular necrosis rate is 40%. Roposch et al^[8]^ analyze 6 related reports of 358 children through Meta and discovered that 19% of the team with ossification center in femoral head are discovered avascular necrosis, while 22% of the team without ossification center in femoral head are discovered avascular necrosis.

Two cases of bilateral avascular necrosis were grade 4 according to Tönnis dislocation classification. Tönnis thinks that there are 2 reasons for avascular necrosis caused by treatment of DDH. One involves intracapsular factors, including the mechanic trauma and the block of nutrient vessels for epiphyseal cartilage during the process of reduction. The other involves extracapsular factors, including blood flow of medial femoral circumflex artery prevented by extreme immobilization position. Ogden^[35]^ proves that compression of iliopsoas to medial femoral circumflex artery or a branch of them causing extracapsular block will lead to AVN with clinical and anatomic results. The 12-month-old child with a severe Tönnis IV dislocation was discovered AVN after the first operation. Since the resistance was high, the mechanic trauma was severe and the iliopsoas was stretched with high tension, the medial femoral circumflex artery of the branch could be compressed, leading to AVN.

**Figure 5:**
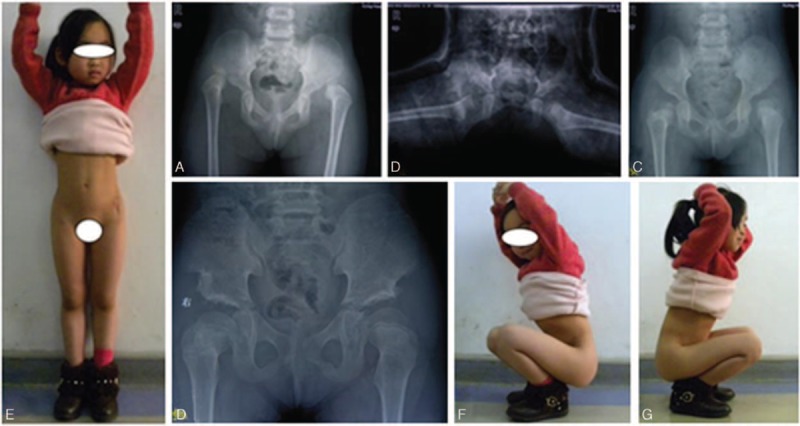
A, 28 months, Tönnis IV bilaterally; AC angle: 45°/46°. B, Plaster cast after reduction. C, Poor reduction (1 year): AC angle: 34°/38°. D, Pemberton acetabuloplasty (1 year). AVN grade I. bilaterally (the 66th month). E–G, Good (Mckay standard).

To our surprise, grade II avascular necrosis was discovered in 1 child who had experienced a secondary Pemberton acetabuloplasty with the spica cast in abduction position of 30°. No impairment of the hip capsule and femoral head blood supply was found in this patient (Fig. 5). We can conclude that persistent and uneven mechanical pressure of acetabulum can lead to avascular necrosis and shape change of femoral head, so proximal femur shortening osteotomy should be applied to reduce the mechanic pressure when necessary.

Among 11 hips of Tönnis grade IV of DDH in our cases, 3 hips were failed in reduction (27.3%) and AVN was discovered in 4 hips (36.4%). The main reason was that the femoral head was mechanically injured since there was high resistance in reduction because of the severe dislocation.

In some cases the Shenton line was discontinous and the femoral head containment was poor, while it was not counted as redislocation. After the thorough discussion, we think discontinous Shenton line and the poor femoral head containment resulted from acetabular developmental dysplasia after the surgery. In our arthroscopic surgery, the joint capsular was not incised and only Pemberton actabuloplasty was applied; therefore, the femoral head containment was good and the Shenton line was continuous.

## 6 Conclusions

From all the above we can conclude that arthroscopic-assisted treatment is an effective way for the therapy of irreducible DDH, which is helpful for the reduction of irreducible DDH of Tönnis I, II, and III, and for the development of acetabular cartilage, coordinated with acetabuloplasty and/or femoral osteotomy when necessary. Compared with open reduction, it has advantages of less trauma and good postoperative function. The iliopsoas release and femoral shortening osteotomy or the preoperative traction can help to reduce the avascular necrosis for the severe dislocation of Tönnis IV.

Things to note are that posterior two-third of outer-rim incision of the labrum is beneficial to the integrity of the inner-rim, which may prevent the redislocation of the femoral head from the labrum.^[28]^ When the articular capsule was exposed, it was incised and the femoral head was rotated anterolaterally to push it to outer flank. Then, the arthroscope was put in the articular cavity and the synovial membrane was resected by articular shaver. We think that the following order is beneficial to the operation technique and vision: the ligamentum teres are cut first, then pulvinar are excised, transverse acetabular ligament is resected afterward, and lastly goes to the labrum.

## Acknowledgments

Great thanks to Professor Jiayong Fan from Department of Foreign Languages of the Fourth Military Medical University for the advice on English writing of this thesis.

## References

[1] Mahan ST Katz JN Kim YJ To screen or not to screen? A decision analysis of the utility of screening for developmental dysplasia of the hip. *J Bone Joint Surg Am* 2009; 91:1705–1719. 19571094 10.2106/JBJS.H.00122 PMC2702253

[2] Phelan N Thoren J Fox C Developmental dysplasia of the hip: incidence and treatment outcomes in the Southeast of Ireland. *Ir J Med Sci* 2015; 184:411–415. 24879336 10.1007/s11845-014-1133-0

[3] Schwend RM Schoenecker P Richards BS Pediatric Orthopaedic Society of North A. Screening the newborn for developmental dysplasia of the hip: now what do we do? *J Pediatr Orthop* 2007; 27:607–610. 17717457 10.1097/BPO.0b013e318142551e

[4] Schoenecker PL Flynn JM Screening for developmental dysplasia of the hip. *Pediatrics* 2007; 119:652–653. 17332226 10.1542/peds.2006-2953

[5] Okano K Yamada K Takahashi K Long-term outcome of Ludloff's medial approach for open reduction of developmental dislocation of the hip in relation to the age at operation. *Int Orthop* 2009; 33:1391–1396. 19449005 10.1007/s00264-009-0800-7 PMC2899135

[6] Pospischill R Weninger J Ganger R Does open reduction of the developmental dislocated hip increase the risk of osteonecrosis? *Clin Orthop Relat Res* 2012; 470:250–260. 21643924 10.1007/s11999-011-1929-4 PMC3237975

[7] Morcuende JA Meyer MD Dolan LA Long-term outcome after open reduction through an anteromedial approach for congenital dislocation of the hip. *J Bone Joint Surg Am* 1997; 79:810–817. 9199376 10.2106/00004623-199706000-00002

[8] Roposch A Stohr KK Dobson M The effect of the femoral head ossific nucleus in the treatment of developmental dysplasia of the hip. A meta-analysis. *J Bone Joint Surg Am* 2009; 91:911–918. 19339576 10.2106/JBJS.H.00096

[9] Byrd JW Jones KS Hip arthroscopy in the presence of dysplasia. *Arthroscopy* 2003; 19:1055–1060. 14673446 10.1016/j.arthro.2003.10.010

[10] Bulut O Ozturk H Tezeren G Arthroscopic-assisted surgical treatment for developmental dislocation of the hip. *Arthroscopy* 2005; 21:574–579. 15891724 10.1016/j.arthro.2005.01.004

[11] Eberhardt O Wirth T Fernandez FF Arthroscopic reduction and acetabuloplasty for the treatment of dislocated hips in children of walking age: a preliminary report. *Arch Orthop Trauma Surg* 2014; 134:1587–1594. 25077783 10.1007/s00402-014-2063-z

[12] Öztürk H Oztemur Z Bulut O Arthroscopic-assisted surgical treatment for developmental dislocation of the hip before the age of 18 months. *Arch Orthop Trauma Surg* 2013; 133:1289–1294. 23728833 10.1007/s00402-013-1781-y

[13] Tonnis D Surgical treatment of congenital dislocation of the hip. *Clin Orthop Relat Res* 1990; 33–40. 2203574

[14] McKay DW A comparison of the innominate and the pericapsular osteotomy in the treatment of congenital dislocation of the hip. *Clin Orthop Relat Res* 1974; 124–132. 4817221 10.1097/00003086-197401000-00013

[15] Severin E Contribution to the knowledge of congenital dislocation of the hip joint. *Acta Chir Scand Suppl* 1941; 84:1–142.

[16] Pemberton PA Pericapsular osteotomy of the ilium for treatment of congenital subluxation and dislocation of the hip. *J Bone Joint Surg Am* 1965; 47:65–86. 14256975

[17] Kalamchi A MacEwen GD Avascular necrosis following treatment of congenital dislocation of the hip. *J Bone Joint Surg Am* 1980; 62:876–888. 7430175

[18] Teng JB Yu CW Wang YZ Sonographic detection of unilateral hip dislocation in a spica cast after closed reduction for developmental dysplasia of the hip. *J Ultrasound Med* 2012; 31:827–831. 22644678 10.7863/jum.2012.31.6.827

[19] Bian Z Guo Y Tian W Causative factors of avascular necrosis after closed reduction for developmental dysplasia of the hip BI. *Chin J Pediatr Surg* 2008; 29:678–681.

[20] Clarke NM Developmental dysplasia of the hip: diagnosis and management to 18 months. *Instr Course Lect* 2014; 63:307–311. 24720316

[21] Wada I Sakuma E Otsuka T The Pavlik harness in the treatment of developmentally dislocated hips: results of Japanese multicenter studies in 1994 and 2008. *J Orthop Sci* 2013; 18:749–753. 23812768 10.1007/s00776-013-0432-z PMC3778211

[22] Ardila OJ Divo EA Moslehy FA Mechanics of hip dysplasia reductions in infants using the Pavlik harness: a physics-based computational model. *J Biomech* 2013; 46:1501–1507. 23631856 10.1016/j.jbiomech.2013.03.031

[23] Atalar H Sayli U Yavuz OY Indicators of successful use of the Pavlik harness in infants with developmental dysplasia of the hip. *Int Orthop* 2007; 31:145–150. 16601983 10.1007/s00264-006-0097-8 PMC2267572

[24] Chen B Yang J Zhang J Early treatment for development dysplasia of the hip using the Pavlik harness. *Chin J Pediatr Surg* 2009; 30:525–528.

[25] Druschel C Placzek R Selka L MRI evaluation of hip containment and congruency after closed reduction in congenital hip dislocation. *Hip Int* 2013; 23:552–559. 24062219 10.5301/hipint.5000070

[26] Shugars RA More RC Arthroscopic hip surgery. *Aorn J* 2005; 82:976. 16478081 10.1016/s0001-2092(06)60250-0

[27] Ilizaliturri VM Jr Chaidez PA Valero FS Hip arthroscopy after previous acetabular osteotomy for developmental dysplasia of the hip. *Arthroscopy* 2005; 21:176–181. 15689866 10.1016/j.arthro.2004.09.011

[28] Chen R Huang L Xu H Clinical study of arthroscopically-assisted treatment for developmental dysplysia of hip in children who unresponed to close reduction. *Chin J Joint Surg* 2008; 2:407–415.

[29] Carreira D Bush-Joseph CA Hip arthroscopy. *Orthopedics* 2006; 29:517–523. 16786944 10.3928/01477447-20060601-07

[30] Ozturk H Bulut O Tezeren G Hip arthroscopy for developmental dislocation. *Orthopedics* 2007; 30:600. 17727011 10.3928/01477447-20070801-06

[31] McCarthy JJ MacEwen GD Hip arthroscopy for the treatment of children with hip dysplasia: a preliminary report. *Orthopedics* 2007; 30:262–264. 17424686 10.3928/01477447-20070401-08

[32] Schoenecker PL Lesker PA Ogata K A dynamic canine model of experimental hip dysplasia. Gross and histological pathology, and the effect of position of immobilization on capital femoral epiphyseal blood flow. *J Bone Joint Surg Am* 1984; 66:1281–1288. 6490704

[33] Cashman JP Round J Taylor G The natural history of developmental dysplasia of the hip after early supervised treatment in the Pavlik harness. A prospective, longitudinal follow-up. *J Bone Joint Surg Br* 2002; 84:418–425. 12002504 10.1302/0301-620x.84b3.12230

[34] Tiderius C Jaramillo D Connolly S Post-closed reduction perfusion magnetic resonance imaging as a predictor of avascular necrosis in developmental hip dysplasia: a preliminary report. *J Pediatr Orthop* 2009; 29:14–20. 19098638 10.1097/BPO.0b013e3181926c40

[35] Ogden JA Changing patterns of proximal femoral vascularity. *J Bone Joint Surg Am* 1974; 56:941–950. 4847241

